# Evaluation of Antimicrobial and Mechanical Properties of a Novel Propolis-Modified Orthodontic Primer: An In-Vitro Study

**DOI:** 10.7759/cureus.46716

**Published:** 2023-10-09

**Authors:** Deepika Katyal, Reshma Mohan, Ravindra Kumar Jain, Shweta Nagesh

**Affiliations:** 1 Orthodontics and Dentofacial Orthopaedics, Saveetha Dental College and Hospitals, Saveetha Institute of Medical and Technical Sciences, Chennai, IND

**Keywords:** mechanical properties, propolis, cytotoxicity, orthodontic primer, antibacterial

## Abstract

Introduction

Accumulation of cariogenic biofilm around the bracket surface and the enamel adhesive interface leads to the formation of white spot lesions which poses an arduous challenge in orthodontics today. The aim of this study was to do a comparative evaluation of the antimicrobial, cytotoxic and mechanical properties of a novel propolis-modified orthodontic primer with a control primer.

Materials and methods

This in-vitro study involved two groups (group A: propolis-modified primer and group B: control primer). Antibacterial properties against *Streptococcus mutans* were evaluated using the agar well diffusion technique to measure the zone of inhibition and mic was evaluated using the two-fold diffusion technique. 3-[4,5-dimethylthiazol-2-yl]-2,5 diphenyl tetrazolium bromide (MTT) fibroblast assay was done to evaluate the cytotoxicity. After bonding brackets on extracted natural teeth (premolars) the shear bond strength (SBS), contact angle (CA) and adhesive remnant index (ARI) were evaluated for both groups. Statistical analysis was done using Statistical Package for the Social Sciences (IBM SPSS Statistics for Windows, IBM Corp., Version 23.0, Armonk, NY), and an independent t-test was performed.

Results

The propolis-modified primer when compared to the control primer had higher zone of inhibition values and lower minimum inhibitory concentration (MIC) values. The MTT fibroblast assay showed that the cell viability % shown by the propolis primer was more than the control primer. There was no statistically significant difference between the two primers for SBS (p>0.05), CA (p>0.05) and ARI (p>0.05) (p=0.05).

Conclusion

The propolis-modified primer showed higher antibacterial activity against *S. mutans* at a lower inhibitory concentration, with less cytotoxicity and no effect on the SBS, CA and ARI scores.

## Introduction

Orthodontic primer is an important agent in the orthodontic bonding system which is used to provide proper bond strength between the bracket and the tooth surface [1]. White spot lesions (WSLs) are caused by the demineralization of subsurface enamel. Enamel demineralization commonly occurs at the juncture between the bracket and tooth surface in a fixed orthodontic treatment [2,3]. Since composite adhesives for orthodontic bonding are applied to the enamel surface after priming, an antimicrobial primer would be beneficial in lowering biofilms and reducing demineralization between the bracket and the tooth surface interface [4]. Adhesion of cariogenic bacteria, like *Streptococcus mutans *(*S. mutans*), is what primarily causes this. Additionally, enamel etching damages the enamel's structure by decalcifying it and increasing the risk of decay [5]. Many attempts have been made to create an antimicrobial orthodontic adhesive system to avoid demineralization at the enamel interface [6,7]. However, the agents used in these studies had some disadvantages such as decreased mechanical properties and short-term release of antimicrobial agents [8,9].

Propolis is a viscous organic material that is gathered by honeybees from numerous plant sources [10]. It has been claimed to have a variety of biological properties, including anti-inflammatory, anti-cancer, antioxidant and anti-fungal properties. Propolis’s antibacterial effect often relates to the action of the flavanone-pinocembrin, flavonoid galangin and caffeic acid, which is dependent on the suppression of bacterial RNA polymerase [11]. Koo et al.'s experiment revealed that propolis prevents *S. mutans* and *Streptococcus sobrinus *(*S. sobrinus*)from growing and adhering to tooth surfaces [12]. The purpose of this in-vitro study was to evaluate different mechanical and antibacterial properties of propolis-modified orthodontic primer and compare it with conventional orthodontic primer.

## Materials and methods

A novel primer containing propolis was tested in this study (Anabond, Stedman Pharma Research, Chennai). According to the manufacturer, the primer contained the ingredients mentioned in Table 1.

**Table 1:** Composition of propolis-modified primer. BisGMA: bisphenol A glycol dimethacrylate, PMGDM: pyromellitic dianhydride glycerol dimethacrylate, HEMA: 2-Hydroxyethyl methacrylate

Component	Percentage
Resin: BisGMA, PMGDM, HEMA	80%
Solvents: Acetone and Ethanol	15%
Additives: Photo initiators, Inhibitors, Stabilisers, etc.	+5%
Propolis	0.1-0.25%

The current study was performed at White Lab, Saveetha Dental College and Hospitals, Chennai, Tamil Nadu. For assessment of the mechanical properties, extracted human premolar and incisor teeth free of any caries or enamel defects were included in the study.

Antibacterial activity

Zone of Inhibition

The agar well diffusion method was used to gauge the primer's antibacterial activity. The tube was incubated at 370 degrees Celsius for 18 hours with the turbidity adjusted to 0.5 McFarland's units after the *S. mutans* bacterial culture was introduced into Brain Heart Infusion (BHI) broth. On the Mueller Hinton Agar plate, the bacterial culture was spread, and using a sterile cork borer, wells were made on the agar plate. Different primer concentrations (50, 40, and 30 µl) were put into each well, and the plate was incubated at 37^o^C for 24 hours. Around each well, the diameter of the zone of inhibition was quantified in millimetres.

Minimum Inhibitory Concentration Assay

The minimum inhibitory concentration (MIC) value of the propolis primer was evaluated using a two-fold dilution method. *S. mutans* strain was grown in a BHI broth at 37 degrees Celsius for 24 h. The MIC was evaluated with different concentrations of the primer ranging from 5,20,175 and 200μl/ml respectively. Dimethyl sulfoxide (DMSO) was used as a vehicle to load the primer into the wells. Sterility control was also maintained. The plate (microtitre plate) was incubated at 37 degrees Celsius for 24 hrs. After 24 h of incubation, in each well a load of 20 µl of Triphenyl Tetrazolium Chloride (TTC) solution was added and the plate was incubated at 37 degrees Celsius for 30 min. The growth of the bacterial pathogens was observed by adding a TTC solution that acts as an indicator. The lowest concentration with no visible growth was observed as MIC.

Cytotoxicity

Different concentrations of primers (0.05%, 0.1%, 0.15%, 0.2%) were treated with DMEM-low glucose media (Dulbecco's Modified Eagle Medium) formulated with 10% fetal bovine serum (FBS) and 1% Penicillin/streptomycin. The media were collected after 24 hrs of immersion and treated with gingival fibroblast cells to test the compatibility. After 24 hrs of culture, 10μL/100mL of MTT (3-[4,5-dimethylthiazol-2-yl]-2,5 diphenyl tetrazolium bromide) reagent (5 mg/mL stock) was added to the cultured cells and then incubated for 4 h to allow the formation of the formazan dye at 37℃. The medium was exchanged for DMSO (200 μL) and was left to stand for 10 minutes. The reaction product was transferred to a 96-well enzyme-linked immunosorbent assay (ELISA) plate and A590 was measured with an ELISA plate reader. Mitochondrial dehydrogenase activity was assessed using MTT assay and cell necrosis was evaluated using flow cytometry (propidium iodide staining).

Thirty non-carious human premolars, which were obtained by extraction from patients who were to undergo orthodontic treatment, were randomly allocated into two groups: Group A - conventional orthodontic primer and Group B - propolis-modified orthodontic primer. Non-fluoridated pumice was used to clean the buccal surfaces of all teeth. After properly cleaning and drying the teeth using a three-way air syringe, the teeth were polished with a rubber cup. Maxillary or mandibular stainless-steel brackets (Koden Basic Series Brackets, Koden Inc., NY) were bonded to the enamel samples with different orthodontic primers.

Shear bond strength (SBS) assessment

The SBS assessment was done using a universal bending test machine (Instron 4465, Canton, Mass., USA) with a crosshead speed of 1 mm/min. A chisel-edged plunger was used to apply an occluso-gingival load to the brackets, creating a shear load at the bracket-tooth interface. The teeth used were extracted premolars.

Adhesive remnant index (ARI) assessment

Afterwards, the same surface of the teeth was evaluated under a scanning electron microscope (SEM) to evaluate the ARI, based on the scoring described by Artun and Bergland [13]. The scoring system of the index is mentioned in Table 2.

**Table 2:** ARI scoring index ARI scoring index by Artun and Bergland (1984) [13]. ARI: adhesive remanent index

Score	Interpretation
0	No adhesive left on tooth
1	Less than half of the adhesive left on the tooth surface
2	More than half of the adhesive left on the tooth surface
3	All the adhesive left on the tooth, with distinct impression of the bracket mesh

The enamel surface was scored as follows: score 0, no composite resin left on the tooth; score 1, less than half of composite resin left on the tooth; score 2, more than half of composite resin left on the tooth; score 3, all composite resin left on the tooth with a distinct impression of the bracket base.

Contact angle (CA) measurements

For the measurement of CA, 30 extracted central incisor teeth were used. These teeth were prepared as described above. The enamel surface was then thinly sliced using a hard tissue microtome. The enamel surfaces mounted were then on a CA goniometer as described by Pandian SM et al. [14], which were then exposed to droplets of the CH primer and conventional primer. These were further subjected to topographic analysis using SEM.

Statistical analysis

The statistical analysis was performed using Statistical Package for the Social Sciences (IBM SPSS Statistics for Windows, IBM Corp., Version 23.0, Armonk, NY). Normality tests were performed using Shapiro-Wilk’s test. Intra-examiner reliability was done using the kappa statistics. Descriptive statistics were performed to obtain the mean and standard deviation of all the measurements. An independent ‘t’ test was performed to compare the means between the two groups with regard to SBS and CA. A chi-square test was done to compare the ARI scores between both groups.

## Results

Antimicrobial properties

Zone of Inhibition

**Figure 1:**
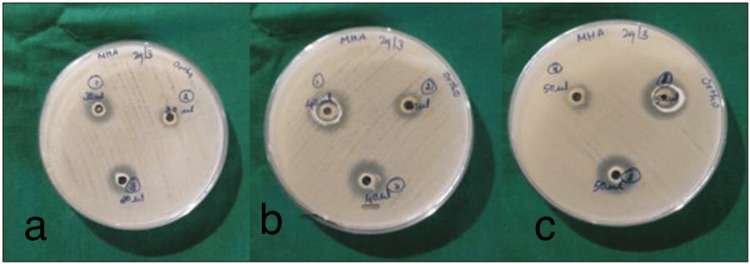
a: Zones of inhibition at 30μl, b: zones of inhibition at 40μl, c: zones of inhibition at 50μl

The preliminary screening of the antimicrobial activity of propolis-modified primer (different concentrations) was assessed in terms of the zone of inhibition of bacterial growth (*S. mutans*). The propolis-modified primer depicted better antibacterial activity at 50, 40 and 30 μl of concentration in comparison to the control primer. The results of antibacterial activities are presented in Table 3 and Figure 1.

**Table 3:** Zone of inhibition of primers. S. mutans: Streptococcus mutans

Antibacterial Activity (Zone of Inhibition)						
Microorganism	Contro primer			Propolis-modified primer		
Concentration in µl	50	40	30	50	40	30
Zone of Inhibition in mm against *S. mutans*	17	15	13	18	15	14

Minimum Inhibitory Concentration

**Figure 2:**
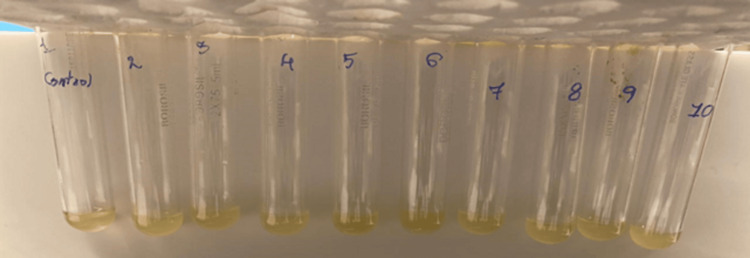
Two-fold dilution technique for evaluating minimum inhibitory concentration.

Propolis-modified primer has potentially inhibited the growth of *S. mutans* at a concentration of 5%, w/v when compared to the conventional primer (10%, w/v) (Figure 2).

Cytotoxicity

The results of the MTT fibroblast assay are depicted in Table 4. The percentage of cell viability of the CH primer was 57+/-7% at 0.05 microlitres, 35+/-7% at 0.1 microliters, 14+/-5% at 0.15 microlitre, 8+/-4% at 0.2 microlitres which was higher when compared to the cell viability of the control primer.

**Table 4:** Comparison of shear bond strength and contact angle between two groups.

Mechanical Properties	Control	Propolis-Modified Primer	P Value
Shear bond strength	5.37+/-3.4 MPa	7.67+/-3.0 MPa	0.10
Contact angle	46.7+/-6.77	33.41+/-10	0.07

Kappa values for intraobserver reliability showed good agreement (0.7 to 1.00). Mean SBS was higher for the propolis-modified primer but no significant intergroup difference was noted (p> 0.05). The results indicated a lower CA with propolis-modified primer but no statistically significant difference between the two primers was seen (p>0.05).

On comparing the ARI scores between the groups, no significant results were obtained (Table 5).

**Table 5:** ARI index scores between the two primers. ARI: adhesive remanent index

ARI Scores	Control	Propolis-Modified Primer	P Value
0	0(0)	0(0)	0.52
1	1(1)	1(0)	
2	2(6)	2(5)	
3	3(8)	3(10)	

Both groups showed a higher percentage of samples with a score of 3.

Group A had 53.3% samples with score-3, 40% with score-2 and 6.67% with score-1. Group B had 66.66% samples with score-3 and 33.33% samples with score-2.

## Discussion

The invention of enamel bonding for use in orthodontic applications in 1965 is regarded as a crucial turning point in orthodontic care. To strengthen the relationship between brackets and teeth surface, new technologies involving unique materials are continually being developed [15-17]. It is widely known that individuals undergoing orthodontic treatment with fixed appliances are more prone to the buildup of bacterial plaque and enamel demineralization [18]. This can be attributed to the difficulty in mechanical removal of the plaque due to the interference of the appliance. One of the most efficient ways to avoid enamel demineralization is by incorporating different materials into composite resins and orthodontic adhesives that are resistant to bacterial adhesion and biofilm formation [19]. The study was conducted to evaluate the mechanical and adhesive properties of propolis-modified primer. Propolis is a non-toxic and safe substance with antibacterial activity [20].

Bond strengths of 5.9 to 7.8 MPa have been demonstrated by Reynolds and von Fraunhofer to be sufficient to withstand chewing forces [21]. The results of the current study, the addition of propolis by 0.1-0.25% to the primer showed an increase in SBS compared to conventional primer. However, this was not statistically significant. A study by Sodagar et al. has shown that the addition of propolis nanoparticles by 1-5% maintains the SBS to an acceptable clinical range. However, a 10% increase in propolis nanoparticles can reduce the SBS significantly lower compared to the conventional adhesive and much lower than the acceptable range, which is not recommended for clinical use [19]. A study by Mirhashemi et al. has shown that the addition of nanoparticles of up to 5% retained the SBS of the adhesive to a clinically acceptable range [22]. In contrast to this, there are few studies that have shown that an increase of nanoparticles from 5 to 10% reduced the SBS of the adhesive material [23,24]. All these studies have shown that an increase of nanoparticles from 1 to 2% was acceptable to retain the SBS of the adhesive material.

The capacity of the primer to adapt and wet the enamel surface affects how well the bracket base will adhere [25]. The CA of the liquid primer can be used to determine its wetting behaviour. The relationship between CA and wettability is inverse, therefore the lower the CA, the better the wettability, and vice versa [26]. The results of the current study showed that the CA of the novel primer was less than the control primer. However, it was not statistically significant (p> 0.05).

Clinicians consider the ARI score when making their choice of orthodontic adhesive [27]. The less adhesive that is left on the enamel surface after debonding, the lower the risk of fracture and damage [28]. According to the current study, there is no statistical difference between the ARI scores between the two groups. Hence, it is revealed that the new primer's addition of propolis had no effect on the bond failure pattern during debonding. Few studies have reported similar results where the addition of nanoparticles did not alter the ARI scores of the test samples post-debonding [24].

Limitations

Limitations of the present study include that the antibacterial properties of the novel primer were only tested against *S. mutans.* Though* S. mutans* is the major contributor to biofilm formation in the oral cavity, the antimicrobial properties against other bacterial and fungal oral organisms can be evaluated. In-vivo studies are warranted to further substantiate the use of the chitosan-modified primer.

## Conclusions

The current study has shown that the novel primer had better antibacterial properties at lower concentrations than the unmodified primer against *S. mutans*. The incorporation of propolis in the primer did not significantly alter the SBS, CA and bond failure patterns in the adhesive material.
